# Glycotherapy: A New Paradigm in Breast Cancer Research

**DOI:** 10.3390/biom12040487

**Published:** 2022-03-23

**Authors:** Dipak K. Banerjee, Arelis Seijo Lebrón, Krishna Baksi

**Affiliations:** 1 Department of Biochemistry, School of Medicine, University of Puerto Rico, Medical Sciences Campus, San Juan, PR 00936-5067, USA; arelisseijo@gmail.com; 2 Department of Anatomy and Cell Biology, School of Medicine, Universidad Central del Caribe, Bayamon, PR 00960-3001, USA; krishna.baksi@uccaribe.edu

**Keywords:** breast cancer, angiogenesis, glycotherapy, asparagine-linked glycoprotein, tunicamycin, *N*-acetylglucosaminyl 1-phosphate transferase, ER stress, unfolded protein response, *upr*

## Abstract

Breast cancer is an ancient disease recognized first by the Egyptians as early as 1600 BC. The first cancer-causing gene in a chicken tumor virus was found in 1970. The United States signed the National Cancer Act in 1971, authorizing federal funding for cancer research. Irrespective of multi-disciplinary approaches, diverting a great deal of public and private resources, breast cancer remains at the forefront of human diseases, affecting as many as one in eight women during their lifetime. Because of overarching challenges and changes in the breast cancer landscape, five-year disease-free survival is no longer considered adequate. The absence of a cure, and the presence of drug resistance, severe side effects, and destruction of the patient’s quality of life, as well as the fact that therapy is often expensive, making it unaffordable to many, have created anxiety among patients, families, and friends. One of the reasons for the failure of cancer therapeutics is that the approaches do not consider cancer holistically. Characteristically, all breast cancer cells and their microenvironmental capillary endothelial cells express asparagine-linked (*N*-linked) glycoproteins with diverse structures. We tested a small biological molecule, Tunicamycin, that blocks a specific step of the protein *N*-glycosylation pathway in the endoplasmic reticulum (ER), i.e., the catalytic activity of *N*-acetylglusosaminyl 1-phosphate transferase (GPT). The outcome was overwhelmingly exciting. Tunicamycin quantitatively inhibits angiogenesis in vitro and in vivo, and inhibits the breast tumor progression of multiple subtypes in pre-clinical mouse models with “zero” toxicity. Mechanistic details support ER stress-induced unfolded protein response (*upr*) signaling as the cause for the apoptotic death of both cancer and the microvascular endothelial cells. Additionally, it interferes with Wnt signaling. We therefore conclude that Tunicamycin can be expected to supersede the current therapeutics to become a glycotherapy for treating breast cancer of all subtypes.

## 1. Introduction

Breast cancer incidence in women has increased from about 1 in 11 women in 1975 to 1 in 8 women (a 12.4% increase) in recent years. This is alarming and has led the breast cancer landscape to become a global health crisis [1]. The disease accounts for nearly a quarter of all cancers combined in women and it is estimated that nearly 2.1 million women are diagnosed with the disease each year [2]. One of the reasons that the number of cancer cases is continuously rising is because the number of women in the age groups at cancer risk is increasing. Invasive breast cancer incidence rates are stable in white women, but they have increased by 0.3% per year in black women. On the other hand, the rates appear to be stable among women <50 years of age, but it have increased among women >50 years of age. The median age at diagnosis is 62 [3].

**Figure 1:**
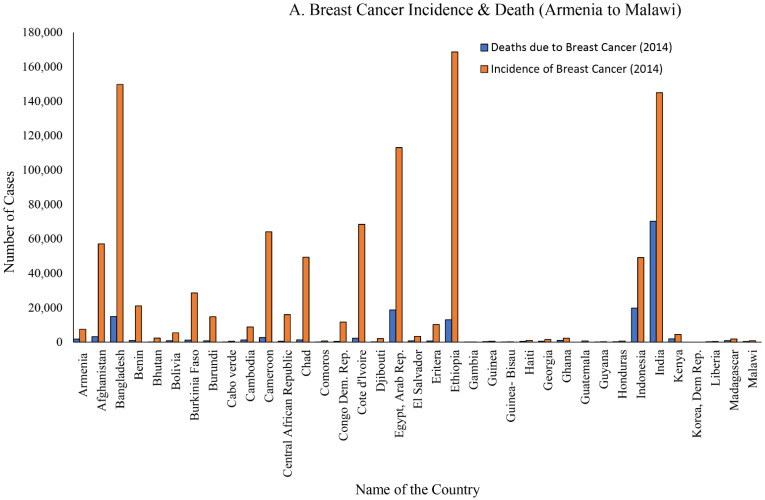

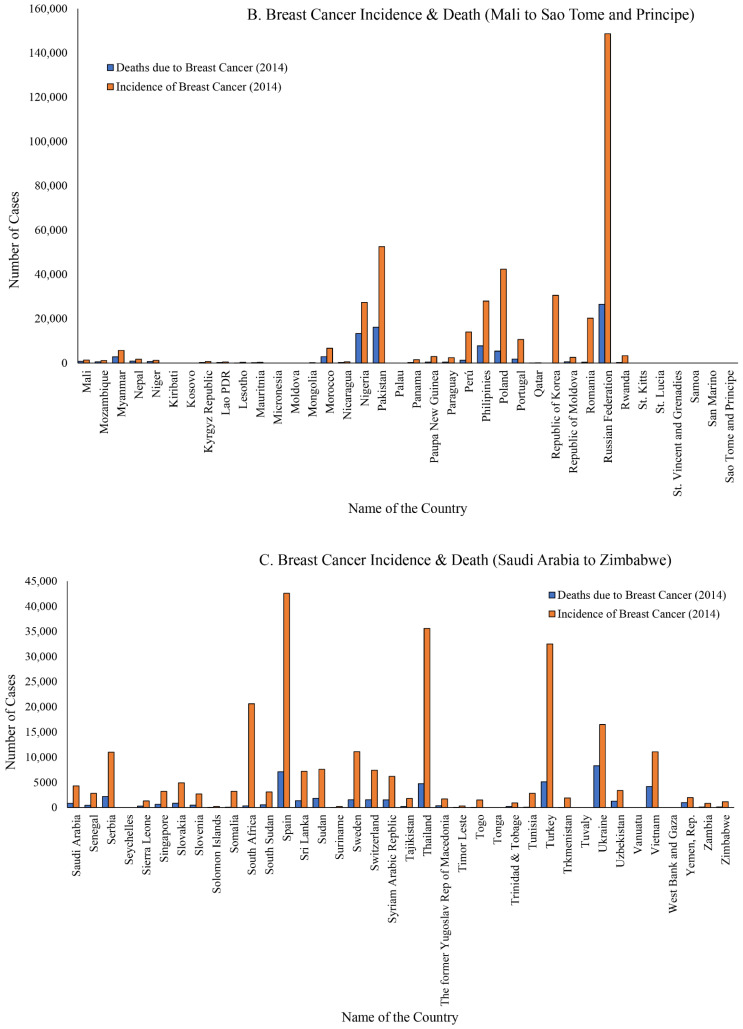
Breast cancer incidence from low- and medium-income countries. The information is collected from each country’s website presented in 2014. (**A**) Breast cancer incidence and death from Armenia to Malawi; (**B**) breast cancer incidence and death from Mali to Sao Tome and Principe; and (**C**) breast cancer incidence and death from Saudi Arabia to Zimbabwe.

The disease has no geographical boundaries, and affects women from low-, medium-, and high-income countries as well as women with diverse ethnic backgrounds (Table 1, Figure 1) [4,5]. In the United States, in 2021 it is estimated that 281,550 new cases of invasive breast cancer were diagnosed, as well as 49,290 new cases of non-invasive breast cancer [1,6].

Breast cancer is classified into four molecular subtypes. Luminal A (ER^+^ and/or PR^+^/HER2^−^) tends to be slow-growing and less aggressive than other subtypes and is associated with the most favorable prognosis, because it is usually responsive to hormonal therapy [7,8]; luminal B (ER^+^ and/or PR^+^/HER2^+^) subtype is characterized clinically as always being positive for HER2 and is also hormone receptor-positive (ER^+^, PR^+^); it tends to be higher grade than luminal A and is also associated with poorer outcomes [7,8]; HER2 overexpressing (ER^−^ or PR^−^/HER2^+^) is characterized clinically by being hormone receptor-negative (ER^−^/PR^−^) and HER2^+^, and has the worst prognosis; however, the widespread use of targeted therapies for HER2^+^ cancers have substantially improved outcomes for these patients [9]; and basal-like (ER^−^/PR^−^/HER2^−^), also called triple-negative because it is hormone receptor-negative (ER^−^/PR^−^) and HER2^−^. The majority (about 75%) of triple-negative breast cancers fall into a basal-like subtype that is defined by gene expression profiling [10]. They have a poorer prognosis than other subtypes because advances in treatment are far behind those for other molecular subtypes [9,11], and are also more common in premenopausal women and those with a *BRCA1* gene mutation [12]. Based on SEER (Surveillance, Epidemiology, and End Results) data, in the United States, 71% of tumors are ER^+^ and/or PR^+^/HER2^−^, 12% are triple-negative (ER^−^/PR^−^/HER2^−^), 12% are ER^+^ and/or PR^+^/HER2^+^, and 5% are ER^−^/HER2^+^. 15% of breast cancers among women with either node-positive disease or metastatic stage IV disease at diagnosis vary by breast cancer subtype. Consequently, the classification and/or the categorization of breast cancer subtypes could play a significant role in treatments and management. The characterization of ER, PR, and/or HER2 expression status is performed on the basis of the immunohistochemistry of tumor tissues. Gene microarray technology may be considered for further advancement.

## 2. Risk Factors

Epidemiologic studies have established several risk factors (physical, hormonal, environmental, and genetic) for breast cancer. These apply at the population level, but it has not been proven whether they are effective in predicting an individual’s risk of breast cancer. Furthermore, analysis of the data collected by the first National Health and Nutrition Examination (NHANES 1) survey and NHEFS suggests that no more than an estimated 41% of breast cancer cases in the United States are attributable to key risk factors identified through this analysis (i.e., later age at first birth, null parity, family history of breast cancer, and higher socioeconomic status). Factors affecting obesity, immunity, and the tumor’s environment within the body, as well as the exogenous environmental exposures, can also influence the development of the disease. Genetic mutations such as mutation of *BRCA1* and *BRCA2* genes also contribute to breast cancer. Most risk factors are not modifiable. However, the amount of lifetime exposure of breast tissue to circulating ovarian hormones is within one’s control and is modifiable. Potentially modifiable breast cancer risk factors include postmenopausal obesity, use of combined estrogen and progestin menopausal hormone-replacement therapy, alcohol consumption [13,14], smoking, and being physically inactive. Ductal cell carcinoma in situ (DCIS) and radiation exposure are also risk factors for breast cancer.

## 3. Current Strategy for Treating Breast Cancer

The bottom line is that before introducing a treatment, breast cancer needs to be diagnosed as accurately as possible based on the biochemical changes that take place not only in tumor cells or tissues, but also in their surroundings. Attention also needs to be paid to the kind of breast cancer and its spread. This may require or involve multiple approaches. Commonly used diagnostic procedures for breast cancer include breast ultrasound (sonograms), diagnostic mammogram, MRI, and biopsies (fine-needle aspiration, core biopsy, or open biopsy). Liquid biopsy is also a possibility.

There were 684,996 breast cancer deaths globally in 2020 [4]. The 2021 estimates for the United States indicate that 43,600 women will die from breast cancer. The median age of breast cancer death is 69 [4]. It is estimated that 1.04 million women will die from breast cancer worldwide in 2040 if no major changes in prevention or treatment are forthcoming [15]. The generalized treatments often used include surgery, chemotherapy, hormonal therapy, biological therapy, radiation therapy, adjuvant therapy, and complementary and alternative medicine.

Most breast cancer deaths are unfortunately due to metastasis and its consequences including impairment of the functions of vital organs like the lung, liver, and brain. Metastatic breast cancer (a stage IV cancer) cells frequently differ from the preceding primary breast cancer cells in terms of properties such as receptor status, and often develop resistance to previous treatment and acquire special properties. There is no single therapeutic strategy that can meet all these challenges and cure metastatic breast cancer. Through standard cancer care, these patients may live longer, but their quality of life is inadequate. The treatments depend on the location of the metastatic tumors and many of them are mentioned above. Hormonal therapy, however, is guided by factors such as (i) characteristics of the cancer cells; (ii) spreading of cancer; (iii) the symptoms; and (iv) past breast cancer treatments. The current approaches of metastatic breast cancer treatment are tyrosine kinase inhibitor (lapatinib); PARP inhibitors with *BRCA1* or *BRCA2* gene mutation; CDK4/6 inhibitor (palbociclib); PI3 kinase inhibitors; and immunotherapy (“checkpoint inhibitors”). The prognosis, however, depends upon the stage at diagnosis: e.g., the 5-year survival rate is 100% for Stage 0, 98% for Stage I, 88% for Stage II, 56% for Stage IIIA, 49% for Stage IIIB, and 16% for Stage IV.

The disease is due to a progressive accumulation of mutations and chromosomal aberrations causing altered growth properties and oncogenic transformations of cells. Both endogenous and exogenous factors contribute to the development and progression of the disease [16]. Furthermore, metastatic load due to epithelial–mesenchymal transition (EMT; [17]) raises further complications. The current therapeutic strategies narrowly target individual cell types (http://www.angiogenesis.org (accessed on 8 November 2021); http://cancernet.nci.nih.gov (accessed on 8 November 2021); www.clinicaltrials.gov (accessed on 8 November 2021)). The Pharmaceutical Research and Manufacturers of America as well as the ClinicalTrials.gov identifies 1683 registered ongoing clinical trials or recruiting for the evaluation of drug interventions for breast cancer. Many clinical trials evaluate either existing drugs in new combinations or at different stages of the disease. This raises another question as to whether the current approaches of developing more drugs and conducting more clinical trials can be redesigned to accelerate the rate of progress, thus ending breast cancer, or has the time come to consider a paradigm shift for developing a new generation of breast cancer therapeutics for curing the disease?

## 4. Quest for a New Therapy

The hallmarks of cancer follow the core principles of sustainability to proliferative signaling, ability to evade growth suppressors, ability to resist cell death, ability to enable replicative immortality, angiogenesis induction, and activation of invasion and metastasis [18,19]. Therefore, elimination of breast cancer requires looking at cancer holistically (i.e., both the tumor microenvironment, including the microvasculature, and the tumor cells). Angiogenesis, i.e., *neo*-vascularization is “key” to breast cancer progression. Because of a symbiotic relationship, tumor cells turn on the “angiogenic switch”, causing endothelial cell migration from pre-existing vasculature, capillary budding, the establishment of capillary loops, and finally *neo*-vascular remodeling. “Angiogenic switch” activators are the tumor microenvironment; mutation in oncogenes or tumor-suppressor genes; pro-angiogenic molecules (VEGF, FGF-2, EGF, PDGF, PIGF, and MMPs); and anti-angiogenic factors (thrombospondin, angiostatin, tumstatin, and endostatin) [20,21]. Based on this information, several products have been suggested and/or developed. Unfortunately, none of them has cured the disease. Second, if they are effective, they are effective only for a small patient population. Third, there are side effects, which compromise the patient’s quality of life, and are also not necessarily affordable to everyone. Therefore, any new product must address these issues.

## 5. Glycosylation and Cancer Malignancy

Aberrant glycosylation occurs in cancers, and many glycosyl epitopes constitute tumor-associated carbohydrate antigens (TACA; [22]). Studies have indicated that aberrant glycosylation is a result of initial oncogenic transformation, as well as a “key” event in the induction of invasion and metastasis [22,23]. High expression of some glycosyl epitopes promotes invasion and metastasis, leading to shorter (5–10 year) survival rates of breast cancer patients [24,25,26]. Meanwhile, the expression of some other glycosyl epitopes suppresses tumor progression, leading to higher post-operative survival rates [27]. The former category of epitopes includes β6GlcNAc branching in *N*-linked glycan structure; sialyl-Tn in O-linked (serine/threonine-linked) structure; sialyl-Le^x^, sialyl-Le^a^, and Le^y^ in either *N*-linked, O-linked, or lipid-linked structure; GM2, GD3, and sialyl-Gb5. The latter category includes β4GlcNAc competitive with β6GlcNAc; histo-blood group A and B competitive with sialylated structures including sialyl-Le^x^ and sialyl-Le^a^; Gb5 competitive with sialyl-Gb5. The expression mechanism of these glycosyl epitopes in terms of the status of their respective glycosyltransferase genes has also been extensively studied [25]. In addition, asparagine-linked (*N*-linked) glycoproteins have also been found to play a critical role in angiogenesis and tumor progression [28,29,30,31,32,33,34,35]. Inhibition of “hybrid” and “complex”-type *N*-glycan synthesis inhibits the formation of capillary tubes. In contrast, inhibiting only “complex”-type *N*-glycan, but not the “hybrid” type, does not inhibit tube formation [31,33]. In addition, sialic acid expression is upregulated in breast cancer and is associated with altered protein sialylation, which contributes to cancer progression and metastasis [36]. Sialic acids belong to a family of nine-carbon monosaccharides that are synthesized in the cytosol and activated in the nucleus [36]. The most common member of the sialic acid family is *N*-acetylneuraminic acid, which is found in glycan chains on cell surface proteins and lipids [37]. Altered sialylation has been implicated in carcinogenesis via mechanisms involving dysregulated cell–cell adhesion, cell–matrix adhesion, and cell migration [37].

Sialic acids can mask cancer cells and impede them from recognition by immune cells, thereby enhancing their survival and proliferation [38]. The negative charge on sialic acid could also enhance repulsion in cancer cells with altered sialylation, resulting in increased cell–cell repulsion in favor of metastasis [39,40]. One study reports blocking sialic acid suppresses tumor growth by enhancing T-cell mediated tumor immunity [37]. On the other hand, hematological parameters are utilized in breast cancer prognosis for the assessment of pathological state and response to therapy [41].

Thus, there is a paradigm shift in current cancer research recognizing the importance of glycosylation as being critical for either promoting or inhibiting the tumor cell invasion and metastasis. This led us to hypothesize that (i) regulation of protein *N*-glycosylation is a “key” to cancer progression; and (ii) understanding the molecular details of the process a new cancer therapy could emerge. We focused our studies on the effect of external and internal stimuli on the regulation of *N*-linked protein glycosylation and its impact on angiogenesis.

## 6. Regulation of Asparagine-Linked (*N*-Linked) Protein Glycosylation and Its Role in Capillary Endothelial Cell Proliferation/Angiogenesis

The *N*-glycans are structurally distinct and evolutionarily conserved. In *N*-linked glycans, the sugars are linked to one another by glycosidic bonds between carbon 1 and 4 of the sugar molecules, whereas the carbohydrate residues are attached to the nitrogen atom of an asparagine side chain by a β-glycosidic linkage present in a consensus tripeptide sequence Asn-X-Ser/Thr (X could be any amino acid except cystine or proline), i.e., the *sequon.* The formation of the glycosidic bond is energetically unfavorable, and the reaction is coupled to the hydrolysis of two molecules of ATP [42]. The energy required for *N*-glycosidic linkage comes from the hydrolysis of a pyrophosphate molecule [42]. In a fully processed *N*-linked glycoprotein, the glycan structures are either “high mannose”, or “hybrid” or “complex” types or a mixture of all three types [43]. The synthesis of *N*-linked glycan in glycome [42] starts at the endoplasmic reticulum (ER), continues through the Golgi, and ends at the plasma membrane for secretion or becomes embedded in the plasma membrane. The biosynthetic process requires (i) synthesis of dolichol-linked precursor oligosaccharide (i.e., lipid-linked oligosaccharide; LLO); (ii) *en bloc* transfer of the precursor oligosaccharide to protein; and (iii) processing of the *N*-glycan chain. Nonetheless, each of these processes is influenced by the cellular *milieu*.

### 6.1. Characterization of a Capillary Endothelial Cell Line

Earlier studies used endothelial cells from the umbilical vein to study angiogenesis in vitro. It is expected that the genetic makeup and/or the function(s) of endothelial cells between capillaries and the veins are different but the similarities and/or the differences have never been addressed. Serendipitously, we have developed an immortal endothelial cell line from the microvasculature of bovine adrenal medulla whose phenotypic and genotypic characters are well preserved till today and turns out to be an excellent model to study angiogenesis in vitro [44]. These cells grow on cell culture plastic surfaces in the absence of special growth factors or collagen overlays and differentiate into organized structures similar to true capillaries. The cells contain Factor VIII:C, a marker for endothelial cells, and form intercellular junctions. They also synthesize and secrete basal lamina structures and engage in transcytosis, a characteristic ultrastructural and functional combination of exocytosis and endocytosis across the thin endothelial cell processes. These cells can also take up catecholamine by a non-neuronal uptake mechanism (Banerjee DK—unpublished observation) and deaminate them by A-type monoamine oxidase, an enzyme functionally distinct from the B-type monoamine oxidase found in neighboring chromaffin cells [44,45,46].

### 6.2. Estrogens Induce Capillary Tube Formation

Abnormal angiogenesis underlies many pathological conditions and is critical for the growth and maintenance of various types of tumors, including hormone-dependent cancers. Estrogens are potent carcinogens in humans and rodents and are involved in regulating angiogenesis. We have examined the effect of estrogen on the behavior of this endothelial cell line. The results demonstrate that 17β-estradiol (E2), at different conditions, exerts both stimulatory and inhibitory effects on endothelial cell adhesion, proliferation, and tube formation in vitro. Utilizing a cellular attachment assay, chronic exposure to nanomolar concentrations of E2 (i.e., 1 and 10 nM) increased endothelial cell adhesion significantly compared to vehicle-treated controls. Cellular adhesion is inhibited by micromolar concentrations of E2. Cell count, PCNA (proliferating cell nuclear antigen) immunohistochemistry, and Western blot analyses demonstrate enhanced cell proliferation at low E2 concentration in an estrogen-depleted medium. Inhibition of cellular proliferation is observed in both estrogen-replete and -depleted medium at higher E2 concentrations (i.e., 1 and 10 µM). Furthermore, in vitro tube formation is increased 3.0-fold in the presence of 10 nM and at higher E2 concentrations [47].

### 6.3. Effect of Microenvironment on Endothelial Cell Behavior

Carbon dioxide, a product of aerobic glycolysis, is largely spent in maintaining the acid–base equilibrium of the cell, but the residual amount (5–10%) must be utilized in maintaining its microenvironment, i.e., the pH, because its deprivation may serve as a signal for cellular dysfunction. Capillary endothelial cells when cultured in the absence of CO_2_ never become attached to the culture dish and die easily. Buffering the culturing media with 10 mM HEPES-NaHCO_3_, pH 7.4, helps attach cells to the petri plate but does not result in capillary-like characteristics being exhibited. The cells synthesize “complex” and “high mannose” types of *N*-linked glycoproteins, and the ratio of [^3^H] mannose to [^14^C] leucine is approximately 3.5-fold higher compared to normally cultured cells, irrespective of a ~32% reduction in protein synthesis. This is primarily due to reduced K_m_ for GDP-mannose for Dol-P-Man synthase (DPMS), a “key” glycosyltransferase for the LLO biosynthesis [28].

### 6.4. The Role of Extracellular Signaling on Endothelial Cell Function

Microvascular endothelial cells are remarkably quiescent cells, but in response to “angiogenic stimuli”, they proliferate, start to invade, and migrate through the surrounding extracellular matrix forming a new capillary network [48]. Although factors derived from normal tissues and factors derived from tumors have been shown to induce angiogenesis [49,50,51,52], the changes in microvascular endothelium causing it to become invasive are poorly understood, because a relevant model of the capillary wall is lacking. We studied two factors, insulin and the activator(s) of intracellular cAMP, on our non-transformed capillary endothelial cell model.

#### 6.4.1. Effect of Insulin

The capillary endothelial cells express high-affinity (K_d_ = 0.04 nM) and low-affinity (K_d_ = 4.7 nM) insulin receptors on their cell surface, with a total number of 210,000 high-affinity receptors per cell. The receptors are functionally coupled, because insulin stimulated 2-deoxyglucose transport across the plasma membrane [53]. When analyzed, the rate of LLO synthesis, as well as its turnover (i.e., t_½_), were increased in the insulin-treated cells. This is corroborated by the enhanced glycosylation of Factor VIII:C [53], an M_r_ 270,000 Da *N*-linked glycoprotein with multiple *N*-glycosylation sites [54]. Insulin treatment supports enhanced *N*-glycosylation, but the process is independent of both intracellular Dol-P level and DPMS gene transcription but is most likely due to increased intracellular level of GDP-mannose. Insulin enhances the cell doubling time, thus blocking angiogenesis. However, making a high level of secretory Factor VIII:C helps provide a fibrin-like matrix for easy differentiation of capillary endothelial cells into capillaries [29,55].

#### 6.4.2. Effect of Intracellular cAMP and the cAMP-Mediated Pathway

The human body is under the influence of the endocrine system. Many of them signal through binding to their specific receptors on the cell surface. The β-adrenergic receptor is a member of the GPCR family of receptors and signals through intracellular cAMP. [^3^H]-Dihydroalprenolol ([^3^H]-DHA) binding to the isolated plasma membrane indicates that the capillary endothelial cells express high-affinity (K_d_ = 0.27 ± 0.09 × 10^−9^ M) and low-affinity (K_d_ = 2.96 ± 0.31 × 10^−9^ M) receptors with the corresponding B_max_ of 5.1 ± 0.05 and 70.0 ± 0.02 pmol/mg protein, respectively. Inhibition of [^3^H]-DHA binding with atenolol (a β_1_-antagonist) and ICI 118,551 (a β_2_-antagonist) suggests that the IC_50cor_ (≈K_i_) for atenolol for the high-affinity site are 0.08 ± 0.03 × 10^−12^ M and 0.25 ± 0.08 × 10^−12^ M, respectively. Displacement of [^3^H]-DHA binding to the endothelial cell plasma membrane by the agonists isoproterenol (a synthetic β-agonist), epinephrine (a natural product and a hormone) and norepinephrine (again a natural product and a hormone) establish a relative order of K_i_ for these agents as isoproterenol (0.56 ± 0.19 × 10^−9^ M) < epinephrine (0.77 ± 0.26 × 10^−9^ M) ≥ norepinephrine (0.71 ± 0.24 × 10^−9^ M) for the high-affinity site. The corresponding values for the low-affinity site are 4.62 ± 0.64 × 10^−9^ M, 6.21 ± 0.86 × 10^−9^ M and 5.90 ± 0.82 × 10^−9^ M, respectively, for the same agonists. Increased intracellular cAMP accompanied with cellular proliferation in the presence of isoproterenol suggests not only the coupling of β-adrenoreceptors to the adenylate cyclase system, but also its involvement in endothelial cell proliferation [56]. Similarly, glucagon, whose second messenger is cAMP, as well as other intracellular cAMP enhancers such as cholera toxin, 8Br-cAMP, etc., not only stimulate capillary endothelial cell proliferation by reducing the cell doubling time, but also help in capillary lumen formation [29,56]. In a synchronized culture when treated with 8Br-cAMP, the G1 phase of the cell cycle is reduced by 12 h and the population doubled after 56 h. Bcl-2, a member of the pro-angiogenic family of proteins remains unchanged so are the caspases-3 and -9, the anti-angiogenic members [57].

The ratio of “high-mannose” to “complex”-type *N*-glycans is a significant determinant for functional modulation. Cells examined by flow cytometry after labeling with FITC-Con A (specificity = α-D-Mannose, α-D-Glucose, or a Branched mannose) and Texas Red-WGA (specificity = GlcNAc-β-(1,4)-GlcNAc)1-4 > β-GlcNAc-NeuAc) indicate the fluorescence intensity for Con A decreases in control cells but increases in 8Br-cAMP-treated cells as the cells continue through the cell cycle. During the same period, the WGA fluorescence intensity rises concomitantly in 8Br-cAMP-treated cells. More specifically, the *N*-glycosylation of capillary endothelial cell marker glycoprotein Factor VIII:C is increased in isoproterenol (1 × 10^−7^ M)-treated cells. To evaluate whether this increase is due to an activation of the protein *N*-glycosylation pathway, the LLO biosynthesis is monitored. The availability of LLO (Glc_3_Man_9_GlcNAc_2_-PP-Dol) is not only essential, it is also a rate-limiting step in *N*-linked protein glycosylation. [2-^3^H]-Mannose incorporation indicates a time-dependent increase in LLO biosynthesis and turn-over in isoproterenol (1 × 10^−7^ M)-treated cells. Other adenylate cyclase activators such as forskolin (1 × 10^−6^ M), cholera toxin (100 ng/mL), and prostaglandin E1 (10 × 10^−6^ M) all support the same event [57,58].

#### 6.4.3. The Status of DPMS

DPMS catalyzes a “key” step in LLO biosynthesis. It elongates Man_5_GlcNAc_2_-PP-Dol to Man_9_GlcNAc_2_-PP-Dol before acquiring three glucose residues. In isoproterenol-treated capillary endothelial cells, there is an increase in Dol-P-Man synthesis. In vitro activity assay of DPMS in microsomes isolated from cells treated with either 8Br-cAMP or isoproterenol indicate a higher catalytic activity for DPMS. When correlated with cellular function, increased DPMS activity has been found to be associated with angiogenesis. The inability of actinomycin D to prevent the enhancement of DPMS activity eliminates the possibility of increased *dpm* gene transcription. cDNA cloning and sequencing of the *dpm* gene identifies the presence of a cAMP-dependent protein kinase (PKA) motif in the DPMS protein sequence [59]. Phosphorylation of microsomal membrane followed by western blotting identifies DPMS as M_r_ 32 kDa phosphoprotein [60]. Interestingly, the PKA motif has been found to be highly conserved and present in all DPMS from protozoan parasites to humans [61].

## 7. Inhibition of Asparagine-Linked Protein Glycosylation and Its Impact on Angiogenesis

The choices are limited when it comes to inhibiting protein *N*-glycosylation. Among the methods available, the majority are of biological origin, and only a few are synthetic molecules. Amphomycin (M_r_ 1290 Da, a lipopeptide with 10 amino acids and a product of *Streptomyces canus*) and Tunicamycin (M_r_ 844.94 Da, a glucosamine-containing pyrimidine nucleoside and a product of *Streptomyces lysosuparificus*) inhibit the early steps of LLO biosynthesis in the ER. Amphomycin binds to dolichol monophosphate (Dol-P) in the presence of Ca^2+^ and inhibits GlcNAc-PP-Dol, Man-P-Dol and Glc-P-Dol synthesis. Tunicamycin, on the other hand, is a competitive inhibitor of *N*-acetylglucosaminyl 1-phosphate transferase (GPT) and inhibits the synthesis of GlcNAc-PP-Dol only. It competes with the substrate UDP-GlcNAc. The presence of a fatty acid side chain makes both of them lipophilic and surface active. When tested, Amphomycin and Tunicamycin inhibit capillary endothelial cell proliferation, i.e., in situ angiogenesis [62].

**Figure 2:**
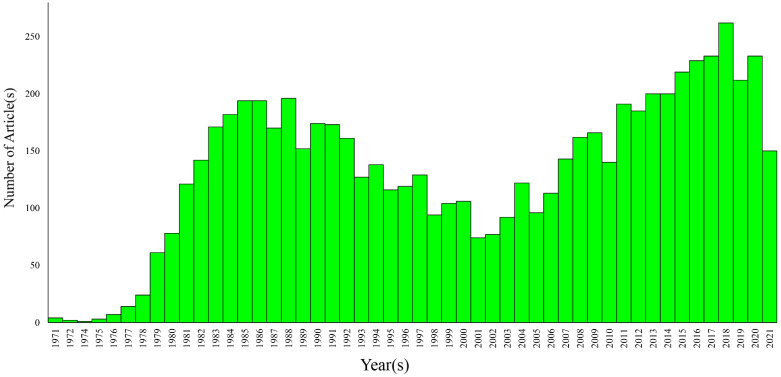
Number of articles published between 1971 and 2021 that used Tunicamycin in all studies. The information was collected from PubMed (https://pubmed.ncbi.nlm.nih.gov (accessed on 26 September 2021)). Search query: Tunicamycin.

**Figure 3:**
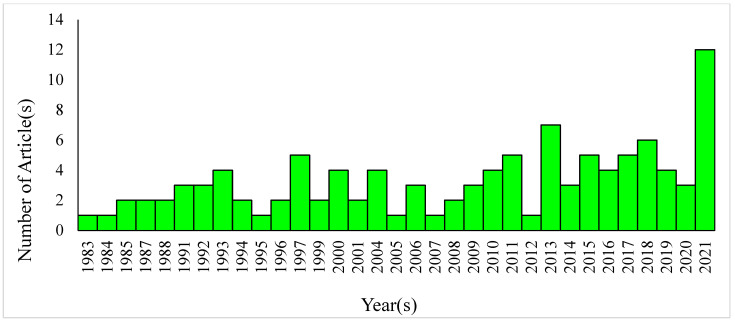
Number of articles published between 1983 and 2021 that used Tunicamycin in breast cancer studies. The information was collected from PubMed (https://pubmed.ncbi.nlm.nih.gov (accessed on 26 September 2021)) Search query: Tunicamycin AND breast cancer.

To avoid the steric hindrance of Amphomycin and its inhibiting multiple steps, we selected Tunicamycin in our studies. Based on PubMed searches, 6820 articles were published between 1971 and 2021 that used Tunicamycin (Figure 2). Similar searches also resulted in 109 articles for the use of Tunicamycin for studying breast cancer (Figure 3).

Tunicamycin (M_r_ 844.94 Da) was first isolated from the soil bacteria *Streptomyces lysosuperificus* [63], and hence has a biological nature. Structurally, it is a glucosamine-containing pyrimidine nucleoside. The original isolate is a mixture of sixteen different homologs with varying molecular weights owing to the variability in fatty acid side chain conjugation. When fractionated and the biological activity is tested for each homolog, only four homologs of Tunicamycin turned out to have protein *N*-glycosylation inhibitory activity [64,65]. Tunicamycin is a competitive inhibitor that inhibits the transfer reaction: UDP-GlcNAc + Dol-P ⬄ GlcNAc-PP-Dol + UMP catalyzed by GPT in the ER. We used Tunicamycin, which blocks *N*-glycosylation. There are no TOX data available on purified Tunicamycin. The SDS provided by the supplier (i.e., Sigma Aldrich now owned by the Merck Group) is from the original Tunicamycin, a mixture of sixteen homologs. This, together with the inadequate experimental design, raises questions about the conclusions made in many of those 7000 publications. The information could be misleading and may prevent further development on the use of Tunicamycin in biological/biomedical sciences, including for the use of breast and other types of cancer. When a synchronized culture of a somatic non-transformed capillary endothelial cell [44] was exposed to Tunicamycin, we made the following observations. Tunicamycin treatment (i) changes the cellular morphology, which exhibits cell shrinkage, loss of cell-to-cell contact, compaction of nuclei showing a condensed pyknotic appearance, and membrane fragmentation by light microscopy; (ii) induces considerable surface blebbing, as observed by scanning electron microscopy; and (iii) reduces cell proliferation. The cumulative effects include ER stress, cell cycle arrest in G1, and the induction of apoptosis [62]. Increased intracellular accumulation of the marker glycoprotein Factor VIII:C (~20% *N*-linked glycans) in Tunicamycin-treated cells suggests an un- or under-glycosylated product [54,62]. The effect is time and concentration dependent. It is worth noting that Tunicamycin inhibits only the proliferative cells, and the effect cannot be washed out or reversed by growth factors such as vascular endothelial growth factor (VEGF). Additionally, Tunicamycin-treated cells express quantitatively less phosphorylated VEGF receptor I and II, inhibit Bcl-2 expression, and block multiple bio-signaling pathways. Thus, suggesting Tunicamycin-treated cells have a point of no return but to death [66].

Breakdown of protein *N*-glycosylation machinery in Tunicamycin-treated cells develops “ER stress”. The molecular signature for the ER stress is the upregulation of GRP78/*Bip* (a glucose-regulated protein/immunoglobulin binding protein) in the ER lumen. Upregulated expression of GRP78 supports unfolded protein response (*upr*) signaling and induction of apoptosis (i.e., programmed cell death). The overwhelming evidence on DNA fragmentation, upregulation of pro-apoptotic caspases-3, -9, and -12, as well as an increase in intracellular free calcium ([Ca^2+^]_i_) all support the ER stress-induced apoptotic cell death. The absence of cytochrome c release indicates no apoptosome formation. Hence, the process is independent of mitochondria and suggests an extrinsic apoptotic pathway.

Caspase-12 belongs to the group I family of caspases. Activation of caspase-12 during apoptosis has been reported in mouse, rat, rabbit, cow, and human cells. There are also reports describing activation of caspase-12 in response to ER stress-mediated apoptosis and the caspase cascade initiated in *upr* [67,68]. This cascade is novel and does not depend on either mitochondria or death receptor activation. Upon activation, caspase-12 translocates from the ER to the cytosol, where it directly cleaves pro-caspase-12, which in turn, activates the effector caspase, caspase-3 [68]. The existence of a mitochondrion- and apoptosome-independent activation of caspase-9 by caspase-12 was further supported by an examination of Apaf1^−/−^ mouse embryonic fibroblasts cells, where the ER stress-mediated apoptotic process remained intact [69]. In these cells inhibition of either caspase-12 or caspase-9 rescued the cells from thapsigargin-induced cell death. By contrast, the ER and mitochondria are linked closely and that Ca^2+^ released from the ER eventually accumulates in mitochondria [70]. In addition, ER stress causes oxidative stress and mitochondrial changes that can be blocked by overexpressing Bcl-2. Besides the transcription- and caspase-mediated cell death pathways, disruption of the Ca^2+^-balance leads to calpain activation, which through the cleavage of Bid and pro-caspase-12 contributes to caspase-9 activation.

## 8. Effect of Tunicamycin on In Vivo Angiogenesis

Tunicamycin, when tested in Matrigel™ implants for 10 days in athymic nude [Balb/c (*nu/nu*)] mice, macroscopic as well as H&E-stained microscopic images of the Matrigel™ sections indicate the absence of microvessels (i.e., blood capillaries). Immunohistochemistry of CD34 and CD144 in Matrigel™ sections also supports their reduced expression and explains the presence of quantitatively few blood vessels in Tunicamycin treated Matrigel™ sections. Enhanced protein and mRNA expression of the endogenous anti-angiogenic factor Thrombospondin-1 (Tsp-1) in Tunicamycin-treated Matrigel™ plugs as well as in Tunicamycin-treated capillary endothelial cells supports inhibition of angiogenesis. The anti-angiogenic effect of Tunicamycin is further supported by the decreased Matrigel™ invasion and chemotaxis of Tunicamycin-treated capillary endothelial cells even when VEGF is present [71].

## 9. Effect of Tunicamycin on Breast Tumor Progression

The inability of VEGF to reverse the anti-angiogenic effect of Tunicamycin both in vitro and in vivo allowed testing of Tunicamycin in humanized breast cancers developed in athymic nude [Balb/c (*nu/nu*)] mice. The study used double-negative (ER^−^/PR^−^/HER2^+^) and triple-negative (ER^−^/PR^−^/HER2^−^) breast cancer pre-clinical mouse models with tumors developed either orthotopically (double-negative, MDA-MB-435) or as xenograft (triple-negative, MDA-MB-231). Mice with double-negative tumor (orthotopic) have received Tunicamycin through intravenous injection (iv) 0.0–1.0 mg/Kg body weight once a week for three weeks, and the mice with triple-negative tumor (xenograft) received Tunicamycin 0.0–0.25 mg/Kg orally twice a week for three weeks. Control mice received the vehicle only. The double-negative tumor (grade III breast adenocarcinoma), when treated with 1 mg/Kg of Tunicamycin, regressed approximately 55% after three weeks, whereas the triple-negative tumor regressed approximately 65% after one week of 0.25 mg/Kg Tunicamycin treatment given orally [71]. These doses are far lower than the FDA-approved dose for the breast cancer drug Taxol. Taxol requires 15–60 times more to match a comparable effect of Tunicamycin. In the control group, the tumor growth is almost doubled in three weeks.

To evaluate *N*-glycan status in breast tumors, the tissue sections from the double-negative breast tumor are stained with Texas-Red-WGA conjugate and examined under a fluorescence microscope. The *N*-glycans in tumor microvessels in untreated controls are stained markedly, but the staining intensity per vessel is reduced almost 50% in Tunicamycin-treated tumors. Tumor cells from untreated control also exhibit positive WGA staining, but the intensity is much reduced in the tumor treated with Tunicamycin. Examination of the paraffin section from the excised tumor after 23 days of Tunicamycin treatment by H&E staining indicates a reduction in microvascular density as the Tunicamycin concentrations are increased from zero to 1 mg/Kg. The mitotic index of tumor cells also declined as a consequence of Tunicamycin treatment. To correlate the tumor growth with cellular markers, the expression of Ki-67 (a cellular proliferation marker) and VEGF (a pro-angiogenic molecule) was analyzed immunohistochemically. The results explain that the expression of both Ki-67 and VEGF in tumors from Tunicamycin-treated mice (1.0 mg/kg) is reduced significantly. This parallels the reduction of microvessel count and the mitotic index, respectively. The mice expressed no behavioral or skeletal toxicity [71]. Accumulating evidence thus unequivocally supports that Tunicamycin could lead the way to developing a dual-action glycotherapy for treating breast cancer in the clinic. It is anti-angiogenic on one hand and anti-tumorigenic on the other hand. Hence, the effect is holistic.

Inhibition of *N*-glycan biosynthesis with Tunicamycin has been found to develop “ER stress” and consequently *upr*-mediated apoptosis [72]. To evaluate whether ER stress also exists in breast tumors when treated with Tunicamycin, both tumor microvasculature and the tumor cells in formalin-fixed paraffin-embedded breast tumor tissue sections were examined. The endothelial cells in tumor microvasculature are identified by staining with anti-CD144 (a marker for endothelial cells) antibody and then stained for the GRP78 (an ER chaperone and the ER stress marker). CD144 and GRP78 co-localize on endothelial cells. CD144-staining of endothelium appears a thin line around the vessel as is the GRP78 in the untreated control. On the other hand, in Tunicamycin-treated tumors, a high expression of GRP78 was observed in tumor microvessels. To understand that it is not an indirect effect because of nutritional deprivation due to reduced blood flow in the tumor, the effect of Tunicamycin is currently being evaluated in multiple subtypes of human breast cancer cells. The preliminary results are encouraging and indicate Tunicamycin inhibits their proliferation (Zhang Z et al.—manuscript under preparation). Thus, from both in vitro and in vivo studies, we conclude that Tunicamycin is not only a novel dual-action glycotherapy for treating breast cancer, but also a therapy that does not discriminate between breast cancer subtypes [71,73].

## 10. Cross-Talk between *N*-Acetylglucosaminyl 1-Phosphate Transferase (GPT) and Dolichyl Monophosphate Mannose Synthase (DPMS)

DPMS catalyzes the reaction Dol-P + GDP-Mannose ⬄ Dol-P-Man + GDP downstream to GPT, and its catalytic product Dol-P-Man is an activator for GPT [74,75,76]. When the DPMS activity is examined in Tunicamycin-treated capillary endothelial cells, the DPMS activity is quantitatively inhibited. The effect is time and dose dependent. It is important to note that Tunicamycin does not have any effect on the catalytic activity of DPMS in a test tube. Therefore, the in-cell inhibition of Dol-P-Man production in Tunicamycin-treated capillary endothelial cells is very intriguing. The reason is not fully understood and needs further investigation.

## 11. Nanoformulation Enhances the Anti-Angiogenic Efficacy of Tunicamycin

Nanoparticles <100 nm evade the immune system’s clearing mechanism long enough to reach the targeted disease tissue efficiently. To evaluate the efficiency and efficacy of nanoformulated Tunicamycin, we developed, characterized a series of gold-conjugated Tunicamycin nanoparticles (i.e., Tunicamycin encapsulated in peptide nanotubes, nanotubes bound to gold nanoparticles (Au NPs) conjugated with Tunicamycin, Tunicamycin conjugated with nanotubes, Au NPs bound to tubes and conjugated with Tunicamycin, and Au NPs conjugated with Tunicamycin) and tested them against the in vitro angiogenesis model of capillary endothelial cells in culture. MTT (3-(4,5-methylthiazol-2-yl)-2,5-dipheyl-tetrazolium bromide) assays indicate that nanoparticles (1 μg/mL) inhibit capillary endothelial cells proliferation, ~50% within one hour of treatment, whereas the native Tunicamycin has no effect. The nanoformulated Tunicamycin blocks the cell cycle progression by inhibiting either cyclin D1 and CDK4, or only the cyclin D1, or the CDK4 expression, as well as the expression of phospho Rb (serine-229/threonine-252). Phosphorylation of p53 at serine-392 is down-regulated but not the total p53. Increased expression of GRP-78/*Bip* identifies “ER stress”. Upregulated expression of phospho-PERK (1.6–5.5-fold) and IRE-1 supports induction of *upr*.

The *upr* aims to restore protein folding homeostasis at the initial phase, while this signal transduction pathway triggers apoptosis if ER stress remains unmitigated. In mammalian cells, the *upr* is mediated by three classes of signaling components: PKR-like ER protein kinase (PERK), activating transcription factor 6 (ATF6), and inositol-requiring enzyme 1 (IRE1). Among the three branches, IRE1 is the most evolutionarily conserved arm from yeast to human. IRE1 has two isoforms: IRE1α and IRE1β; IRE1α is widely expressed in most cells and tissues, while IRE1β is restricted to intestinal epithelial cells.

IRE1α is a 100 kDa type I ER-membrane-resident protein consisting of a luminal domain, a transmembrane domain, and a cytoplasmic region. The cytoplasmic region contains a kinase domain and an endoribonuclease (RNase) domain, making IRE1α a bifunctional enzyme. The luminal domain of IRE1α acts as a sensor of the ER unfolded protein load. Combining with the ER chaperone binding immunoglobulin-protein (*Bip*, also termed GRP78), the luminal domain stays in an inactive monomeric state. The activation of this luminal fragment depends on the dissociation with *Bip*, rather than direct interaction with unfolded proteins. As for the transmembrane domain, very little is known about its functional and structural roles thus far. The kinase domain of IRE1α serves as a substrate for IRE1α trans-autophosphorylation, and provides an ATP-binding pocket. Autophosphorylation of the kinase domain and binding of ADP (or ATP in vivo) allosterically regulate dimerization/oligomerization and lead to IRE1α RNase activation.

IRE1α is the most sensitive of the three *upr* branches that are triggered to cope with ER stress in mammalian cells. IRE1α signaling is quite a context-specific on account of many adaptor and modulator proteins that directly interact with it, including heat shock proteins (HSPs), RING finger protein 13 (RNF13), poly (ADP-ribose) polymerase 16 (PARP16/ARTD15), Bax/Bak, and Bax inhibitor-1 (BI-1). The activated IRE1α triggers different downstream pathways depending on the UPRosome formed by distinct modulator proteins. At the initial phase of ER stress, the IRE1α-XBP1 axis functions as an adaptive response. While ER stress sustains or intensifies, signals shift to apoptotic responses. Furthermore, IRE1α signaling can be exploited to the development of a wide range of prevalent human diseases, with cancer being the most characterized.

PARPs were found to regulate DNA damage repair and the cytoplasmic stress response [77]. Human PARP16 (also known as ARTD15) is a tail-anchored ER transmembrane protein with a cytosolic catalytic domain. The luminal C-tail of PARP16 is required for its function, indicating that stress signals may be transduced from the ER domain to the cytosolic domain [78]. During ER stress, PARP16 enzymatic activity is upregulated. Co-immunoprecipitation assay demonstrated a robust association between PARP16 and IRE1α both in the presence and absence of ER stress. The ADP-ribosylation caused by PARP16 increases the kinase and RNase activities of IRE1α. Under ER stress, PARP16 could facilitate *Bip* dissociation from IRE1α. This effect was impaired in PARP16 knockdown, accompanied by an increase of cell death, suggesting PARP16 is involved in cell survival [79].

Down-regulated expression of caspase-9 and caspase-3 suggests a non-canonical pathway of cell death by nanoformulated Tunicamycin [80]. Thus, the take-home message is that nanoformulated Tunicamycin prevents capillary proliferation, which in turn reduces the nutrient flow to the tumor and consequently causes their death by starvation.

## 12. Tunicamycin Interferes with Wnt Signaling and Inhibits Angiogenesis

**Figure 4:**
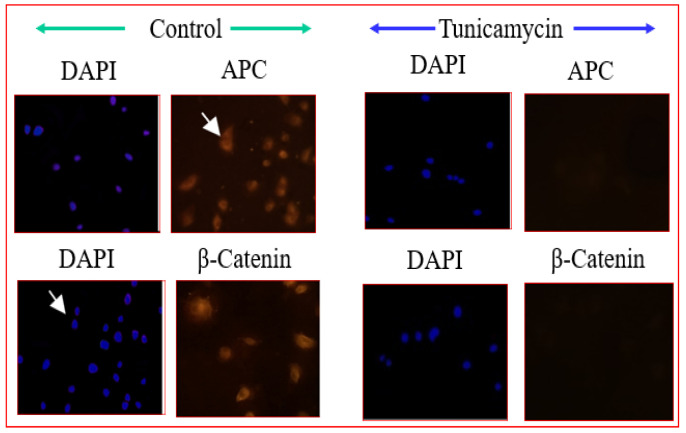
Immunofluorescence microscopy of Wnt proteins: The capillary endothelial cells were cultured for 24 h and synchronized for 72 h. The cells were treated with or without Tunicamycin (1 μg/mL) for 32 h, fixed with 90% ice-cold methanol for 30 s, permeabilized with Triton^™^ X-100 for 10 min, washed with PBS, pH 7.4 3 times, blocked with 3% BSA in PBS, pH 7.4 for 30 min, treated with anti-β-catenin, and anti-APC antibodies (1:50; *v*/*v*) overnight followed by Rhodamin-conjugated rabbit anti-mouse IgG (red) secondary antibody (1:100; *v*/*v*) before collecting the images in a Zeiss Axioskop 2. The nucleus was stained with Hoechst dye (blue).

**Figure 5:**
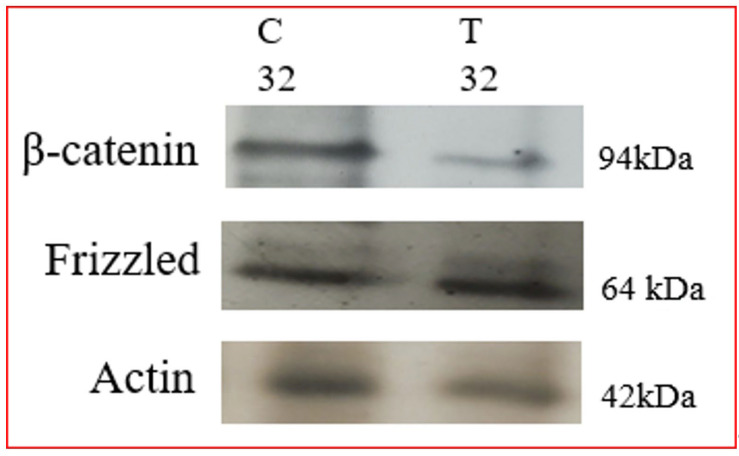
Western blotting of Wnt proteins: The capillary endothelial cells were cultured for 24 h and synchronized for 72 h. The cells were treated with or without Tunicamycin (1 μg/mL) for 32 h. At the end, the proteins were separated on 10% SDS-PAGE and transferred to nitrocellulose membranes. The blots were treated with anti-β-catenin and anti-frizzled monospecific antibodies followed by HRP-conjugated secondary antibodies and developed with EC reagents.

Wnts are a family of secreted growth factors involved in cellular differentiation to organogenesis, and have been suggested to function in angiogenesis. Chemically, they are glycoproteins whose accumulation in the extracellular matrix activates pathways in adjacent cells. Wnt ligands bind to Frizzled receptor, a member of a family of seven-pass transmembrane proteins [81]. A co-receptor, LRP5/6 is required to activate the Wnt/β-catenin and Planar Cell Polarity (PCP) pathways which can be blocked when LRP5/6 is bound to a secreted regulatory protein, Dickkopf-1. Frizzled is a G protein-coupled receptor [82], and Wnt binding to frizzled can activate more than one distinct branch of the Wnt signaling cascade. The different branches of the Wnt signaling pathway are designated the “canonical” signaling pathway, which is referred to as the Wnt/β-catenin signaling pathway, and the “non-canonical”, which includes Wnt signaling through Ca^2+^, PCP and other signaling mechanisms that do not involve β-catenin. In canonical Wnt signaling, the level of cytosolic β-catenin is transiently activated by activating Disheveled (Dvl) to block the phosphorylation of β-catenin by GSK-3. Stabilization of β-catenin promotes its nuclear translocation where it interacts with a family of transcription factors Lef/Tcf [83], activating Wnt/β-catenin target genes, including vascular endothelial growth factor A (VEGF-A), a stimulator of angiogenesis. Therefore, it is ideal to evaluate whether the Wnt pathway is affected in Tunicamycin-treated capillary endothelial cells. The results are expected to provide insight into whether a canonical or a non-canonical pathway is active under the experimental conditions. We used immunofluorescence microscopy and Western blotting as tools, and detected the presence of Frizzled, APC, and β-catenin in the capillary endothelial cell model. Treating cells for 32 h with Tunicamycin causes downregulation of β-catenin expression (Figure 4 and Figure 5). The results thus support that Tunicamycin interferes with the canonical branch of the wnt signaling and impacts angiogenesis.

## 13. Conclusions

**Figure 6:**
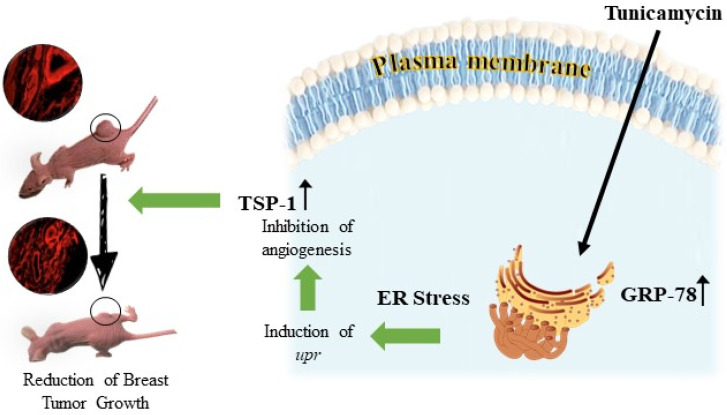
Graphical summary of Tunicamycin action on breast tumor reduction in a pre-clinical mouse model.

Breast tumors require holistic study (meaning the tumor cells and their microenvironment) before it is possible to assign a strategy that could cure the disease even with targeted therapy. The failures to achieve the expected goal with current approaches are manifold, primarily because the intended therapeutics target either the tumor cells or the tumor microvasculature, but not both. It has been recognized that angiogenesis is a “key” to tumor progression without which the tumor will not grow, irrespective of the mutation(s) present in the tumor cells. It will therefore be beneficial if therapeutics are able to find a target common to both tumor cells and the endothelial cells of the tumor microvasculature. While studying the regulation of asparagine-linked protein glycosylation in a capillary endothelial cell line, we documented that protein *N*-glycosylation inhibitors effectively block angiogenesis in vitro. When expanded, Tunicamycin (which has a single cellular target) is an excellent inhibitor for both in vitro and in vivo angiogenesis. Human breast cancer cells do express asparagine-linked glycoproteins irrespective of the cancer subtypes. We used Tunicamycin in pre-clinical mouse models of double- and triple-negative breast tumors to test whether it would inhibit tumor progression. Our observations with tumor tissue sections indicate that the *N*-glycans are reduced both in tumor cells as well as in the tumor microvascular endothelial cells, in parallel to the reduction of tumors upon Tunicamycin treatment. Both tumor cells and the microvasculature experience “ER stress”. Translating the information to individual cell types supports the activation of multiple signaling pathways, leading to a “biological tsunami” with the outcome of apoptotic cell death. Thus, Tunicamycin sets the stage for glycotherapy treating breast cancer in the clinic (Figure 6).

## 14. Future Direction

The use of Tunicamycin in our study was intended to provide a proof of concept for developing a glycotherapy for breast cancer. The mechanistic details of the carefully crafted study and testing in pre-clinical mouse models suggest Tunicamycin as an excellent glycotherapy. Behavioral and/or cytoskeletal toxicities were not detected. The generated information may thus serve as background for an IND (Investigational New Drug) application to the FDA (Food and Drug Administration) to initiate a clinical trial. Additionally, the approaches may be taken to develop other products without disturbing the nucleus of the Tunicamycin structural core.

## Figures and Tables

**Table 1:** Source: GLOBOCAN 2018.

Region	Incidence per 100,000 Females	Mortality per 100,000 Females
Australia/New Zealand	94.2	12.6
Western Europe (Belgium)	92.6	15.5
Northern Europe	90.1	14.1
Northern America	84.8	12.6
Southern Europe	80.3	13.3
Micronesia/Polynesia	58.2	19.1
South America	56.8	13.4
Eastern Europe	54.5	15.5
Caribbean	50.2	18.1
Melanesia (Fiji)	49.7	25.5
Northern Africa	48.9	18.4
Southern Africa	46.2	15.6
Western Asia	45.3	13.6
Eastern Asia	39.2	8.6
Central America	38.3	10.1
South-Eastern Asia	38.1	14.1
Western Africa	37.3	17.8
Eastern Africa	29.9	15.4
Middle Africa	27.9	15.8
South-Central Asia	25.9	13.6

## Data Availability

Data is contained within the article.

## Abbreviations

BIP: immunoglobulin binding protein; cAMP: 3′,5′-cyclic adenosine monophosphate; DCIS: ductal cell carcinoma in situ; DHA: dihydroalprenolol; DPMS: dolichol phosphate mannosyl transferase; EGF: epidermal growth factor; EMT: epithelial-mesenchymal transition; ER: endoplasmic reticulum; FGF: fibroblast growth factor; GPCR: G-protein-couple receptor; GPT: *N*-acetylglusosaminyl 1-phosphate transferase; GRP: glucose responsive protein; LLO: lipid-linked oligosaccharide/dolichol-linked oligosaccharide; MMP: matrix metalloproteinase; MTT: 3-(4,5-methylthiazol-2-yl)-2,5-dipheyl-tetrazolium bromide; PCNA: proliferating cell nuclear antigen; PCP: planar cell polarity; PDGF: platelet derived growth factor; PIGF: placental growth factor; PKA: cAMP-dependent protein kinase; SEER: surveillance, epidemiology, and end results; TACA: tumor-associated carbohydrate antigens; UPR: unfolded protein response; VEGF: vascular endothelial growth factor.
